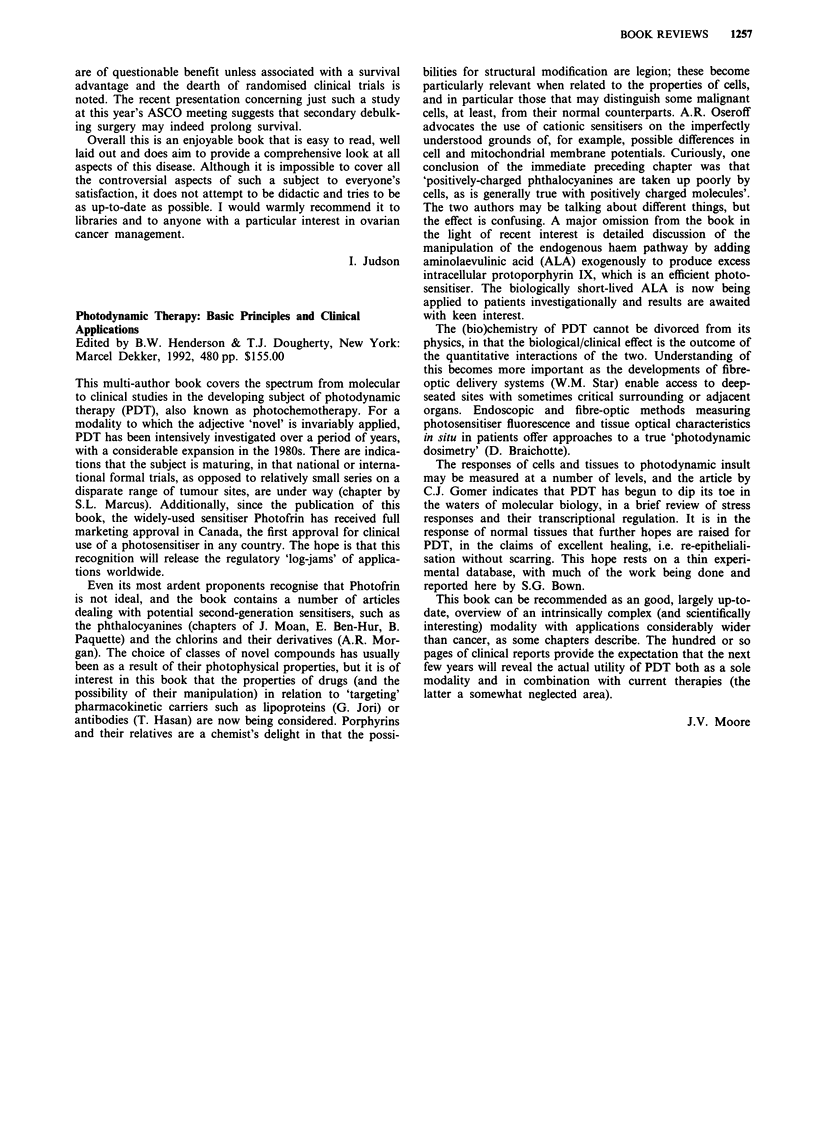# Photodynamic Therapy: Basic Principles and Clinical Applications

**Published:** 1993-12

**Authors:** J.V. Moore


**Photodynamic Therapy: Basic Principles and Clinical Applications**

Edited by B.W. Henderson & T.J. Dougherty, New York: Marcel Dekker, 1992, 480 pp. $155.00

This multi-author book covers the spectrum from molecular to clinical studies in the developing subject of photodynamic therapy (PDT), also known as photochemotherapy. For a modality to which the adjective 'novel' is invariably applied, PDT has been intensively investigated over a period of years, with a considerable expansion in the 1980s. There are indications that the subject is maturing, in that national or international formal trials, as opposed to relatively small series on a disparate range of tumour sites, are under way (chapter by S.L. Marcus). Additionally, since the publication of this book, the widely-used sensitiser Photofrin has received full marketing approval in Canada, the first approval for clinical use of a photosensitiser in any country. The hope is that this recognition will release the regulatory 'log-jams' of applications worldwide.

Even its most ardent proponents recognise that Photofrin is not ideal, and the book contains a number of articles dealing with potential second-generation sensitisers, such as the phthalocyanines (chapters of J. Moan, E. Ben-Hur, B. Paquette) and the chlorins and their derivatives (A.R. Morgan). The choice of classes of novel compounds has usually been as a result of their photophysical properties, but it is of interest in this book that the properties of drugs (and the possibility of their manipulation) in relation to 'targeting' pharmacokinetic carriers such as lipoproteins (G. Jori) or antibodies (T. Hasan) are now being considered. Porphyrins and their relatives are a chemist's delight in that the possibilities for structural modification are legion; these become particularly relevant when related to the properties of cells, and in particular those that may distinguish some malignant cells, at least, from their normal counterparts. A.R. Oseroff advocates the use of cationic sensitisers on the imperfectly understood grounds of, for example, possible differences in cell and mitochondrial membrane potentials. Curiously, one conclusion of the immediate preceding chapter was that 'positively-charged phthalocyanines are taken up poorly by cells, as is generally true with positively charged molecules'. The two authors may be talking about different things, but the effect is confusing. A major omission from the book in the light of recent interest is detailed discussion of the manipulation of the endogenous haem pathway by adding aminolaevulinic acid (ALA) exogenously to produce excess intracellular protoporphyrin IX, which is an efficient photosensitiser. The biologically short-lived ALA is now being applied to patients investigationally and results are awaited with keen interest.

The (bio)chemistry of PDT cannot be divorced from its physics, in that the biological/clinical effect is the outcome of the quantitative interactions of the two. Understanding of this becomes more important as the developments of fibre-optic delivery systems (W.M. Star) enable access to deep-seated sites with sometimes critical surrounding or adjacent organs. Endoscopic and fibre-optic methods measuring photosensitiser fluorescence and tissue optical characteristics *in situ* in patients offer approaches to a true 'photodynamic dosimetry' (D. Braichotte).

The responses of cells and tissues to photodynamic insult may be measured at a number of levels, and the article by C.J. Gomer indicates that PDT has begun to dip its toe in the waters of molecular biology, in a brief review of stress responses and their transcriptional regulation. It is in the response of normal tissues that further hopes are raised for PDT, in the claims of excellent healing, i.e. re-epithelialisation without scarring. This hope rests on a thin experimental database, with much of the work being done and reported here by S.G. Bown.

This book can be recommended as an good, largely up-to-date, overview of an intrinsically complex (and scientifically interesting) modality with applications considerably wider than cancer, as some chapters describe. The hundred or so pages of clinical reports provide the expectation that the next few years will reveal the actual utility of PDT both as a sole modality and in combination with current therapies (the latter a somewhat neglected area).

J.V. Moore